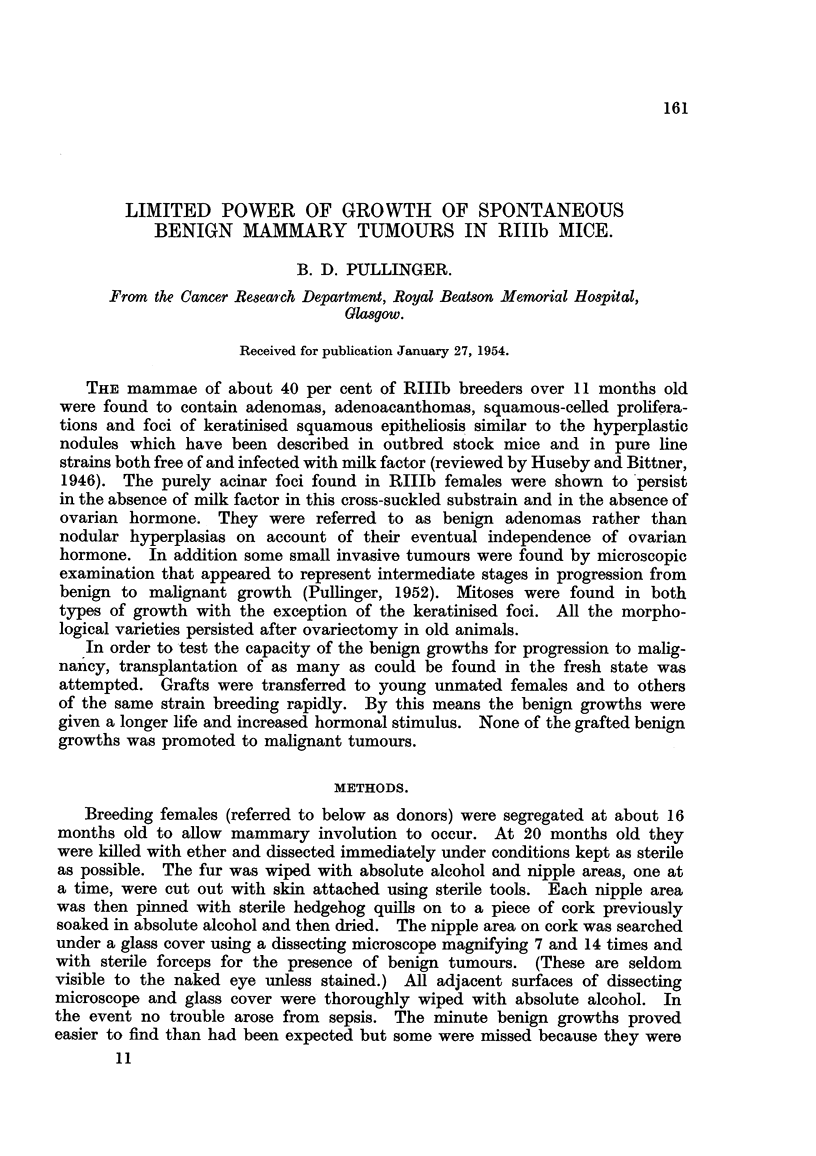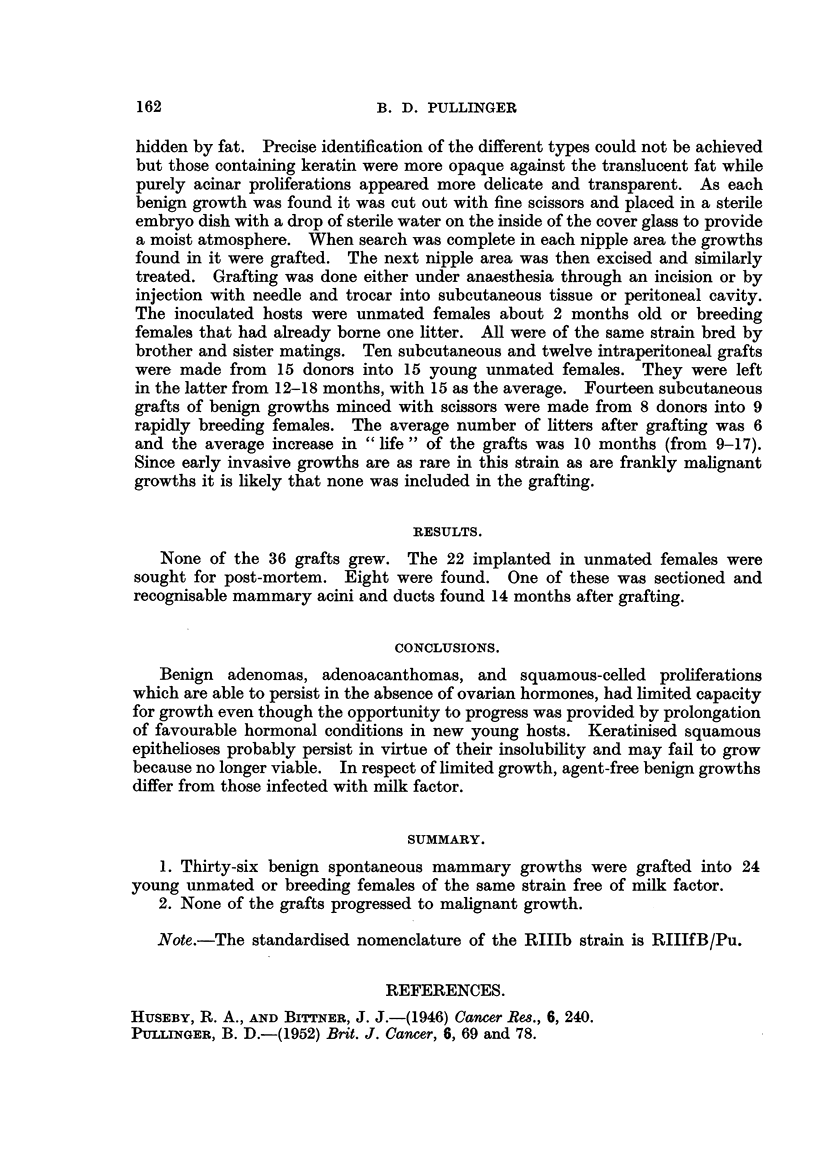# Limited Power of Growth of Spontaneous Benign Mammary Tumours in RIIIb Mice

**DOI:** 10.1038/bjc.1954.14

**Published:** 1954-03

**Authors:** B. D. Pullinger


### LIMITED POWER OF GROWTH OF SPONTANEOUS BENIGN MAMMARY TUMOURS IN RIIIb MICE.

B. D. PULLINGER.

*From the Cancer Research Department, Royal Beatson Memorial Hospital, Glasgow.*

Received for publication January 27, 1954.

THE mammae of about 40 per cent of RIIIb breeders over 11 months old were found to contain adenomas, adenoacanthomas, squamous-celled proliferations and foci of keratinised squamous epitheliosis similar to the hyperplastic nodules which have been described in outbred stock mice and in pure line strains both free of and infected with milk factor (reviewed by Huseby and Bittner, 1946). The purely acinar foci found in RIIIb females were shown to persist in the absence of milk factor in this cross-suckled substrain and in the absence of ovarian hormone. They were referred to as benign adenomas rather than nodular hyperplasias on account of their eventual independence of ovarian hormone. In addition some small invasive tumours were found by microscopic examination that appeared to represent intermediate stages in progression from benign to malignant growth (Pullinger, 1952). Mitoses were found in both types of growth with the exception of the keratinised foci. All the morphological varieties persisted after ovariectomy in old animals.

In order to test the capacity of the benign growths for progression to malignancy, transplantation of as many as could be found in the fresh state was attempted. Grafts were transferred to young unmated females and to others of the same strain breeding rapidly. By this means the benign growths were given a longer life and increased hormonal stimulus. None of the grafted benign growths was promoted to malignant tumours.

### METHODS.

Breeding females (referred to below as donors) were segregated at about 16 months old to allow mammary involution to occur. At 20 months old they were killed with ether and dissected immediately under conditions kept as sterile as possible. The fur was wiped with absolute alcohol and nipple areas, one at a time, were cut out with skin attached using sterile tools. Each nipple area was then pinned with sterile hedgehog quills on to a piece of cork previously soaked in absolute alcohol and then dried. The nipple area on cork was searched under a glass cover using a dissecting microscope magnifying 7 and 14 times and with sterile forceps for the presence of benign tumours. (These are seldom visible to the naked eye unless stained.) All adjacent surfaces of dissecting microscope and glass cover were thoroughly wiped with absolute alcohol. In the event no trouble arose from sepsis. The minute benign growths proved easier to find than had been expected but some were missed because they were hidden by fat. Precise identification of the different types could not be achieved but those containing keratin were more opaque against the translucent fat while purely acinar proliferations appeared more delicate and transparent. As each benign growth was found it was cut out with fine scissors and placed in a sterile embryo dish with a drop of sterile water on the inside of the cover glass to provide a moist atmosphere. When search was complete in each nipple area the growths found in it were grafted. The next nipple area was then excised and similarly treated. Grafting was done either under anaesthesia through an incision or by injection with needle and trocar into subcutaneous tissue or peritoneal cavity. The inoculated hosts were unmated females about 2 months old or breeding females that had already borne one litter. All were of the same strain bred by brother and sister matings. Ten subcutaneous and twelve intraperitoneal grafts were made from 15 donors into 15 young unmated females. They were left in the latter from 12–18 months, with 15 as the average. Fourteen subcutaneous grafts of benign growths minced with scissors were made from 8 donors into 9 rapidly breeding females. The average number of litters after grafting was 6 and the average increase in " life " of the grafts was 10 months (from 9–17). Since early invasive growths are as rare in this strain as are frankly malignant growths it is likely that none was included in the grafting.

### RESULTS.

None of the 36 grafts grew. The 22 implanted in unmated females were sought for post-mortem. Eight were found. One of these was sectioned and recognisable mammary acini and ducts found 14 months after grafting.

### CONCLUSIONS.

Benign adenomas, adenoacanthomas, and squamous-celled proliferations which are able to persist in the absence of ovarian hormones, had limited capacity for growth even though the opportunity to progress was provided by prolongation of favourable hormonal conditions in new young hosts. Keratinised squamous epithelioses probably persist in virtue of their insolubility and may fail to grow because no longer viable. In respect of limited growth, agent-free benign growths differ from those infected with milk factor.

### SUMMARY.

1. Thirty-six benign spontaneous mammary growths were grafted into 24 young unmated or breeding females of the same strain free of milk factor.

2. None of the grafts progressed to malignant growth.

*Note.*—The standardised nomenclature of the RIIIb strain is RIIIfB/Pu.

### REFERENCES.

Huseby, R. A., and Bittner, J. J.—(1946) *Cancer Res.*, **6**, 240.

Pullinger, B. D.—(1952) *Brit. J. Cancer*, **6**, 69 and 78.